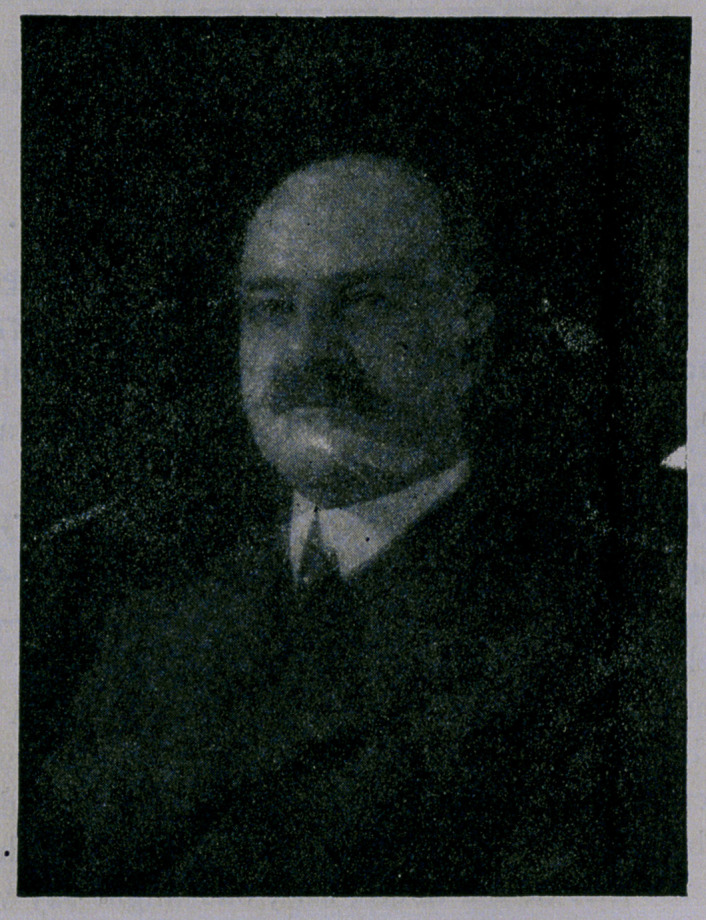# Frank G. Ryan Elected President of Parke, Davis & Co.

**Published:** 1907-06

**Authors:** 


### Frank G. Ryan Elected President of Parke, Davis & Co.

The presidency of Parke, Davis & Co., left vacant by the death of Theodore D. Buhl, has been filled by the advancement of Vice-President and Secretary Frank G. Ryan—an announcement which will be greeted with pleasure by Mr. Ryan's numerous friends throughout the country.

Mr. Ryan was so ideally equipped for this great position that he began to march towards it with what is now seen to have been almost predestination, as soon as he joined fortunes with the house seven years ago. He left the faculty of the Philadelphia College of Pharmacy in the spring of 1900 to become Chief Pharmacist of Parke, Davis & Co. At the end of three years he had made himself so valuable in the councils of the house that he was elected to membership on the Board of Directors. A year and a half later he was given the important post of secretary. Six months later still he was elevated to the vice-presidency. And now, after barely another year, he is given the very highest position within the gift of the house, and, one might say without fear of contradiction, the greatest and most responsible position yet created in the drug trade of the country.

Born in 1861 in Marcellus Falls, New York, Mr. Ryan was educated in the public schools of Elmira, and then spent three years in the well-known pharmacy of Brown & Dawson in Syracuse. In 1882 he entered the Philadelphia College of Pharmacy and was graduated two years later at the age of 23. Two or three years were next spent in various Philadelphia stores, and then he was made assistant professor of pharmacy in his Alma Mater. In 1898 he was given charge of the course in commercial training then established in the P. C. P., and in the meantime he had been made lecturer on pharmacy in the Woman's Medical College of Philadelphia. In June, 1900, Professor Ryan resigned all his connections in Philadelphia and went into the house of Parke, Davis & Co.

The secret of a man's success is never easily analyzed, but it may be said of Frank G. Ryan that he represents that rare, that ideal combination of technical knowledge and experience on the one hand, and business grasp and executive ability on the other. These qualities are all but incompatible, and he who unites them successfully has discovered a philosopher's stone. As president of Parke, Davis & Co., Mr. Ryan will be capable of understanding thoroughly every scientific detail of the vast business now confided to his care, and he will also exhibit that larger vision and that greater capacity for administration which will carry the house forward to conquests even more brilliant than those which have been registered in the past.

Mr. Ryan, accompanied by his daughter Helen, had returned from a seven months' trip around the world only a week or two before his election to the presidency. His main object was to further the interests of his house in Japan, China, and India, but he also visited Manila, Ceylon, Egypt, Paris and London. In Manila an agency was established, which adds another to the considerable list of foreign branches now conducted by the house. In London, on his way back, Mr. Ryan was the guest of honor at two banquets attended by men prominent in British pharmacy and medicine, and when he landed in New York he was greeted at a large reception held at the house of Dr. Jokichi Takamine.

## Figures and Tables

**Figure f1:**